# Effect of jabuticaba (*Plinia jaboticaba*) extract on mead fermentation by *Saccharomyces cerevisiae* and volatile composition

**DOI:** 10.1007/s42770-026-01914-y

**Published:** 2026-04-22

**Authors:** Ludmilla Sousa Lopes, Isabella Bassoto Xavier, Verônica Ortiz Alvarenga, Bruno Gonçalves Botelho

**Affiliations:** 1 Department of Chemistry, ICEx, Universidade Federal de Minas Gerais, Avenida Antônio Carlos 6627, Pampulha, Belo Horizonte, MG 31270-901 Brazil; 2 Department of Food, Faculty of Pharmacy, Universidade Federal de Minas Gerais, Avenida Antônio Carlos 6627, Pampulha, Belo Horizonte, MG 31270-901 Brazil

**Keywords:** Melomel, Yeast assimilable nitrogen, *Saccharomyces cerevisiae*, Non-conventional nutrients, Multivariate analysis

## Abstract

The nutritional deficiency of honey, particularly in yeast assimilable nitrogen (YAN), represents a major limitation for mead production, often resulting in sluggish fermentation and accumulation of undesirable metabolites. This study evaluated the jabuticaba (*Plinia jaboticaba*) aqueous extract as a natural nutritional adjunct for mead fermentation. Musts containing 0 (control), 10, 20, and 30% (v/v) extract were fermented with *Saccharomyces cerevisiae* M05 and characterized using kinetic modeling, physicochemical analysis, and volatile profiling by GC-FID. The supplementation increased YAN from 47.21 to 137.15 mg N/L, reduced fermentation time from 21 to 9 days, and elevated ethanol content from 5.60% to 8.40% (v/v). Principal Component Analysis revealed metabolic modulation characterized by suppression of stress markers (acetic acid, acetaldehyde) and enhanced biosynthesis of Ehrlich pathway-derived higher alcohols. Odor Activity Values indicated that 3-methyl-1-butanol (OAV = 5.93) and 2-phenylethanol (OAV = 3.64) were the primary aroma contributors in supplemented meads, conferring malty and floral notes. Methanol levels remained well within safe limits. The 20% formulation achieved optimal balance between fermentative efficiency and aromatic complexity. Jabuticaba extract proved effective as a clean-label alternative to synthetic supplements.

**Graphical Abstract:**

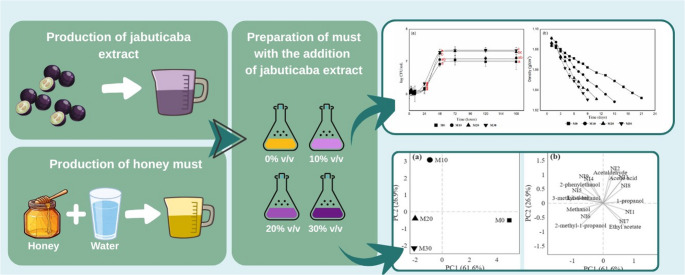

## Introduction

Mead production has expanded globally, driven by the demand for high-value artisanal fermented beverages [1]. However, mead production faces a primary challenge arising from the nutritional composition of honey. Although rich in fermentable sugars (70–80% w/w) [2], honey is naturally deficient in essential nutrients for *Saccharomyces cerevisiae* metabolism, notably yeast assimilable nitrogen (YAN), as well as minerals, amino acids, vitamins, and phenolic compounds [3, 4].

This deficiency is a major limiting factor, frequently resulting in sluggish or arrested fermentation kinetics, low ethanol yield, and biosynthesis of undesirable metabolites, such as volatile sulfur compounds and higher alcohols at suprathreshold concentrations [4]. These effects compromise both process stability and the sensory quality of the final product, underscoring the need for must nutritional supplementation strategies [5].

Conventional industrial practice employs inorganic additives, with diammonium phosphate (DAP) and ammonium sulfate being the most commonly used [4, 6]. However, this approach increases operational costs and contradicts the trend toward clean-label products, characterized by the absence of synthetic additives [7]. In this context, alternative nitrogen sources, such as agroindustrial by-products and plant matrices, have attracted increasing scientific attention [8]. Studies have demonstrated that cowpea extracts [9], rice bran (*Oryza sativa*), and soybean (*Glycine max*) meal [10] correct nutritional deficiencies and accelerate fermentation kinetics. Nevertheless, these investigations are predominantly limited to evaluating process efficiency, neglecting the characterization of the volatile compound profile of the final product.

Fruit-supplemented meads have been investigated using distinct and often fragmented methodological approaches. Certain studies prioritized nutritional and fermentative aspects, such as research with acerola [11] reporting reduced fermentation time attributed to the nutritional contribution of the pulp, and investigations with coconut milk [12] evaluating cell growth dynamics and yeast viability. Other investigations involving pitaya [13], cranberry [14], blackberry [15], and hibiscus combined with birch sap [16] focused on the physicochemical and sensory characterization of the product without correlating them with microbiological indicators. This methodological dichotomy highlights a gap in the literature: most studies address either yeast microbiological behavior or beverage chemical composition, but rarely integrate both approaches. This limitation compromises the understanding of how fruit constituents simultaneously modulate the fermentative performance and the aromatic profile of mead.

Jabuticaba (*Plinia jaboticaba*), a fruit native to the Brazilian Atlantic Forest belonging to the Myrtaceae family, represents a suitable substrate for this purpose [17, 18]. Its composition comprises free amino acids [19], directly related to YAN, as well as minerals such as potassium, magnesium, and zinc, which act as enzymatic cofactors in central metabolic pathways of alcoholic fermentation [20]. Additionally, the fruit’s high perishability and substantial post-harvest losses support its use as a fermentative substrate, offering a strategy for valorizing native resources while mitigating agri-food waste [17, 21].

Although jabuticaba has been employed in alcoholic fermentations, such as fruit wine production, no studies have investigated its use in mead from a perspective that integrates microbiological, physicochemical, and volatile composition parameters.

This study evaluated the use of jabuticaba aqueous extract, produced from whole fruits, as a multifunctional adjunct in mead fermentation. Formulations containing 0% (control), 10%, 20%, and 30% (v/v) extract were prepared, and the effects of supplementation were investigated through kinetic modeling, viable cell enumeration (CFU/mL), physicochemical parameter determination, volatile compound quantification by gas chromatography with flame ionization detection (GC-FID), and calculation of odor activity values (OAV). This approach evaluated the potential of jabuticaba as a nutritional source and aromatic profile modulator, while contributing to the establishment of an integrated methodological strategy applicable to supplemented mead studies.

## Materials and methods

### Raw materials

A total of 15 kg of Sabará Jabuticaba fruits (*Plinia jaboticaba* (Vell.) Berg) were harvested in the municipality of Brumadinho, Minas Gerais, Brazil (coordinates: -20.187352; -44.010481). Fruits were harvested between September and October, corresponding to the peak harvest season in the region. Fruits were manually picked directly from the tree, discarding those that had fallen or with visible physical damage to minimize exogenous microbial load and ensure raw material integrity. Fruit selection prioritized specimens at full physiological maturity, as indicated by peel coloration ranging from dark purple to black, thereby ensuring uniformity in physicochemical composition. This study was registered in the National System for the Management of Genetic Heritage and Associated Traditional Knowledge (SisGen) under number A96E0A3.

Floral honey, predominantly from wildflower sources, was purchased from local commerce in Belo Horizonte, Minas Gerais, Brazil, and used as received without additional processing.

The commercial yeast *Saccharomyces cerevisiae* strain M05 (Mangrove Jack’s) was selected for its specific formulation for mead fermentation. According to manufacturer information, this strain exhibits high ethanol tolerance, high attenuation capacity (95–100%), fermentation temperature range between 15 and 30 °C, and production of floral and fruity esters, characteristics that favor the production of beverages with complex aromatic profiles.

### Preparation and treatment of the must

#### Extract preparation

After harvesting, fruits were selected, sanitized in a 0.03% sodium hypochlorite solution for 15 min, and rinsed with filtered water. An aqueous extraction procedure was adopted to enhance the recovery of soluble compounds and to standardize the total soluble solids content.

Fruits were divided into two fractions: 70% depulped (pulp and seeds) and 30% whole fruits (pulp, seeds, and peels), a proportion selected to prevent excessive astringency from tannins and anthocyanins. Fractions were combined with 7.5 L of filtered water (2:1 w/v ratio), macerated, and heated to boiling to reduce microbial load and promote extraction. The process was conducted until reaching 18 °Brix, confirmed by refractometry after filtration. The extract was stored at − 18 °C as a single batch to minimize experimental variability. Physicochemical characterization is presented in Table 1.

**Table 1:** Physicochemical characterization of jabuticaba extract

Parameter	Value
pH	2.63 ± 0.06
Total titratable acidity (g citric acid/100 g)	1.26 ± 0.05
Total soluble solids (°Brix)	18.00 ± 0.06
Moisture (g/100 g)	81.75 ± 0.17
Total sugars (g/100 g)	17.65 ± 0.55
Reducing sugars (g/100 g)	16.65 ± 0.40

#### Fermentation preparation

Fermentations were conducted in BPA-free polypropylene buckets (5 L capacity, 4 L working volume) equipped with airlock valves.

Must was prepared by diluting honey (84 °Brix) with water at 44 °C to achieve 22 °Brix, followed by addition of jabuticaba extract. Formulations were divided into four treatments: control (0% v/v extract), 10%, 20%, and 30% (v/v), designated M0, M10, M20, and M30. Total soluble solids were adjusted with honey to maintain 22 °Brix across all treatments.

Yeast was rehydrated according to manufacturer’s instructions with volumes adjusted for 4 L: 1.7 g of yeast was added to 35 mL of water (50 °C, cooled to 40 °C), allowed to stand for 15 min, homogenized, and held for 5 additional minutes. Must aliquots (17 mL) were gradually incorporated at 5-minute intervals for thermal acclimatization. The suspension was inoculated into the must, resulting in approximately 10⁶ CFU/mL initial concentration.

**Fig. 1:**
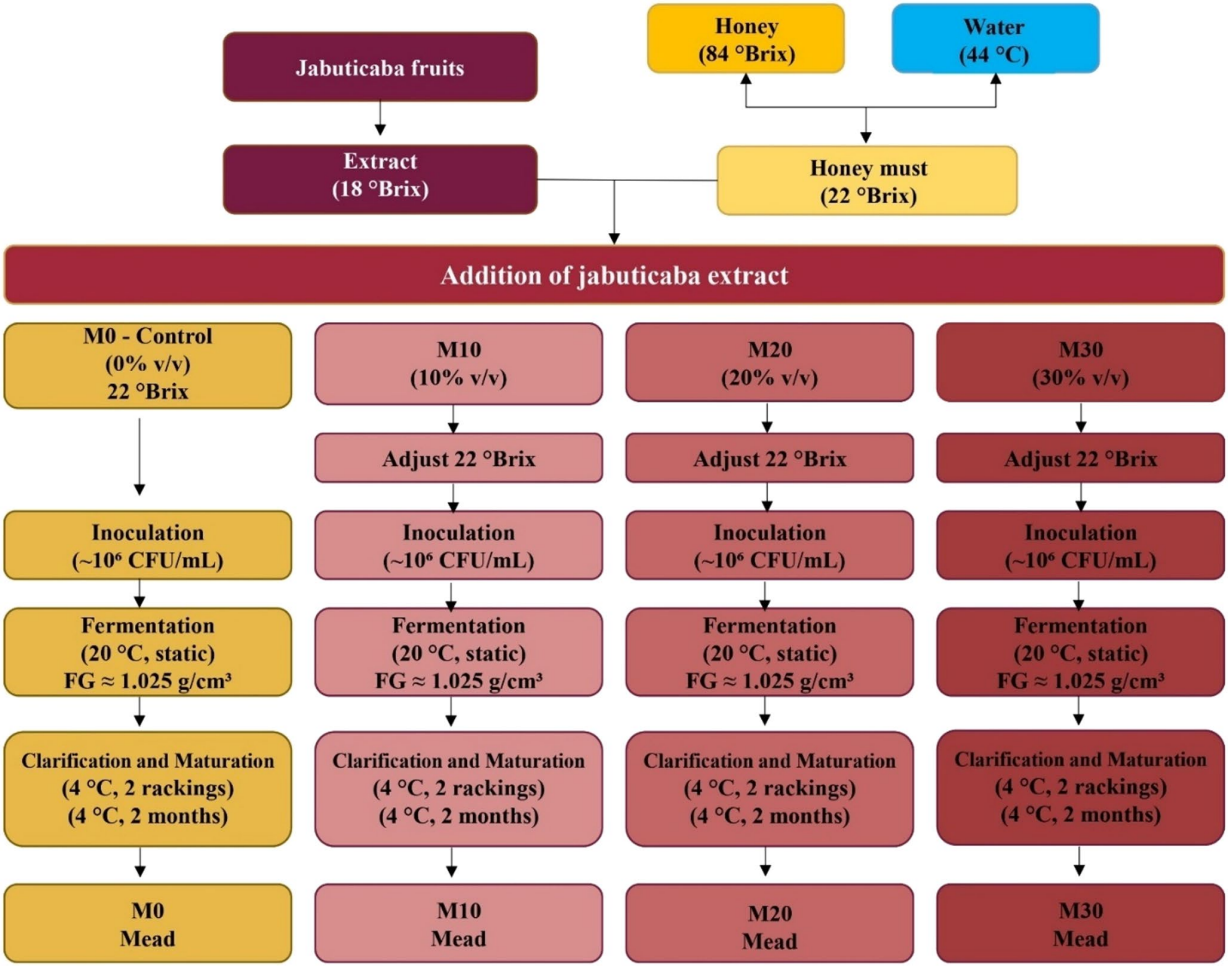
Schematic flow diagram of the mead production process supplemented with jabuticaba extract, including extract preparation, must formulation, fermentation, clarification, and maturation stages. Treatments: M0 (control, 0% v/v), M10 (10% v/v), M20 (20% v/v), and M30 (30% v/v)

Fermentations proceeded at 20 °C under static conditions without aeration and terminated upon reaching approximately 1.025 g/cm³ density, aiming to obtain sweet mead with residual sugar. After fermentation, meads were clarified at 4 °C through two rackings and matured for two months. The process is illustrated schematically in Fig. 1.

### Fermentation monitoring

#### Monitoring of must density and pH

Every 24 h, the musts were gently homogenized. Density and pH were analyzed until 21 days of fermentation. Samples were collected every 24 h. All analyses were performed in triplicate (*n* = 3). The pH analysis was carried out using a JKI^®^ digital pH meter, model JKPHM-005 (Shanghai, China), with a two-point calibration using buffer solutions of pH 4.00 and 7.00.

#### Fermentation kinetics

Cell growth was monitored every 24 h until stationary phase stabilization (168 h). Samples were subjected to serial dilution (10⁻¹ to 10⁻⁹) in 0.1% (w/v) peptone water. Aliquots of 10 µL from each dilution were inoculated in triplicate onto agar plates containing formulated YM agar [malt extract (3 g/L), yeast extract (3 g/L), peptone (5 g/L), glucose (10 g/L), and agar (20 g/L)] using the drop plate technique. Plates were incubated at 30 °C for 48 h for colony counting.

Kinetic parameters, specifically maximum specific growth rate (µ_max_), lag phase (λ), initial log CFU/mL (y₀), and final log CFU/mL (y_max_), were estimated by nonlinear fitting of experimental data to the Baranyi and Roberts model [22] using DMFit software (ComBase). Comparison of kinetic parameters between treatments was based on 95% CI, with non-overlapping intervals adopted as the criterion for statistically significant difference.

### Physicochemical characterization

Physicochemical analyses were performed in triplicate. The total soluble solids content (°Brix) was measured using the refractometric method with a Brix ATC^®^ refractometer at 20 °C. The parameters of total acidity, fixed acidity, volatile acidity, and dry extract were determined according to the methodologies described by the Adolfo Lutz Institute [23].

The total chloride content was determined according to the potentiometric method described by the Instituto Adolfo Lutz [23]. A 20 mL aliquot of the sample was transferred to a 50 mL beaker equipped with a magnetic stirrer and a conductivity electrode. The titration was performed by successive additions of 100 µL of 0.1 mol/L silver nitrate solution. Conductivity was corrected according to Eq. 1:

1 $$\begin{array}{l}Corrected\;conduc\tan ce=\\Conduc\tan ce\ast\frac{\left(Added\;volume+Sample\;volume\right)}{Sample\;volume}\end{array}$$

The volume of titrant at the equivalence point was determined by the intersection of the two regression lines obtained from the titration curve. Total sugar content in the mead samples was determined using the colorimetric method with 3,5-dinitrosalicylic acid (DNS), as described previously [24]. Initially, 500 µL of the sample was hydrolyzed with 2 mL of 2 mol/L HCl in a water bath for 10 min. The solution was then neutralized with 2 mL of NaOH, after which 50 µL of the neutralized sample was diluted in 450 µL of distilled water and mixed with 500 µL of DNS reagent. The mixture was heated for 10 min, followed by the addition of 4 mL of distilled water. Absorbance was measured at 540 nm using a Cary 60 UV-Vis spectrophotometer (Agilent Technologies, USA), and total sugar content was calculated using a glucose standard curve.

### Ethanol quantification by GC-FID and Sugar conversion efficiency

Ethanol concentration in mead samples was quantified using a Shimadzu GC-17 A gas chromatograph equipped with a flame ionization detector (FID) and a Supelcowax 30 capillary column (30 m × 0.53 mm × 2 μm). For sample preparation, 100 µL of pentanol (1% in water) was added as an internal standard, and 50 µL of the sample was injected. Hydrogen was used as the carrier gas at a flow rate of 2.2 mL/min. The injector and detector were maintained at 200 °C. The oven program started at 120 °C and increased to 200 °C at 20 °C/min. Injections were performed in split mode (1:10) with a volume of 0.2 µL. Ethanol concentrations were calculated using calibration curves prepared with ethanol standard solutions.

The efficiency of sugar conversion to ethanol (SCE) was estimated as the ratio between the final ethanol content and the initial sugar concentration in the medium, considering the theoretical maximum yield of 0.511. This approach has been applied in previous fermentation studies [25]. The calculation was performed according to Eq. 2:

2 $$SCE\left(\%\right)=\frac{\left(alcohol\;content\;\left(\%{\displaystyle\frac VV}\right)\ast100\right)}{\left(media\;sugar\;content\;\left(\%\right)\ast0.511\right)}$$

### Yeast assimilable nitrogen (YAN)

YAN values were determined using the formaldehyde method described previously [26]. A 10 mL aliquot of the sample was diluted with 15 mL of distilled water in a 50 mL beaker. The pH was adjusted to 8.1 with 0.1 mol/L NaOH, followed by the addition of 2.5 mL of formaldehyde solution previously adjusted to pH 8.1. After 5 min, the pH was readjusted to 8.1 with 0.05 mol/L NaOH. The assimilable nitrogen concentration was calculated according to Eq. 3:

3 $$YAN\;\left(mg/L\right)=\frac{{Vol}._{\left(NaOH\right)}\ast Conc._{\left(NaOH\right)}\ast14\ast1000}{\left(Vol._{sample}\right)}$$

### Identification of volatile compounds by gas chromatography with flame ionization detection (GC-FID)

Volatile compounds were analyzed using a Shimadzu GC-17 A gas chromatograph equipped with a flame ionization detector (FID) and a Supelcowax column (30 m × 0.53 mm × 2 μm). Hydrogen was used as carrier gas at 2.2 mL/min. For sample preparation, 80 µL of mead was homogenized with 20 µL of 0.1% (v/v) pentanol solution as internal standard. Data acquisition and peak integration were performed using GC Solution software (Shimadzu). The oven program started at 60 °C (7 min hold), ramped to 110 °C at 15 °C/min (5 min hold), then to 200 °C at 5 °C/min (5 min hold), totaling 38.3 min. Compound identification was performed by comparing retention times with authentic standards (Sigma-Aldrich). Quantification was based on external calibration curves. Analytical parameters are presented in Table 2.

Reproducible chromatographic peaks without available commercial standards were coded as NI.1 through NI.9. Only peaks with a coefficient of variation below 10% across triplicates and detected in all samples were included. Although chemical identity was not confirmed due to absence of GC-MS coupling, these compounds were included in PCA to preserve volatile profile complexity.

**Fig. 2:**
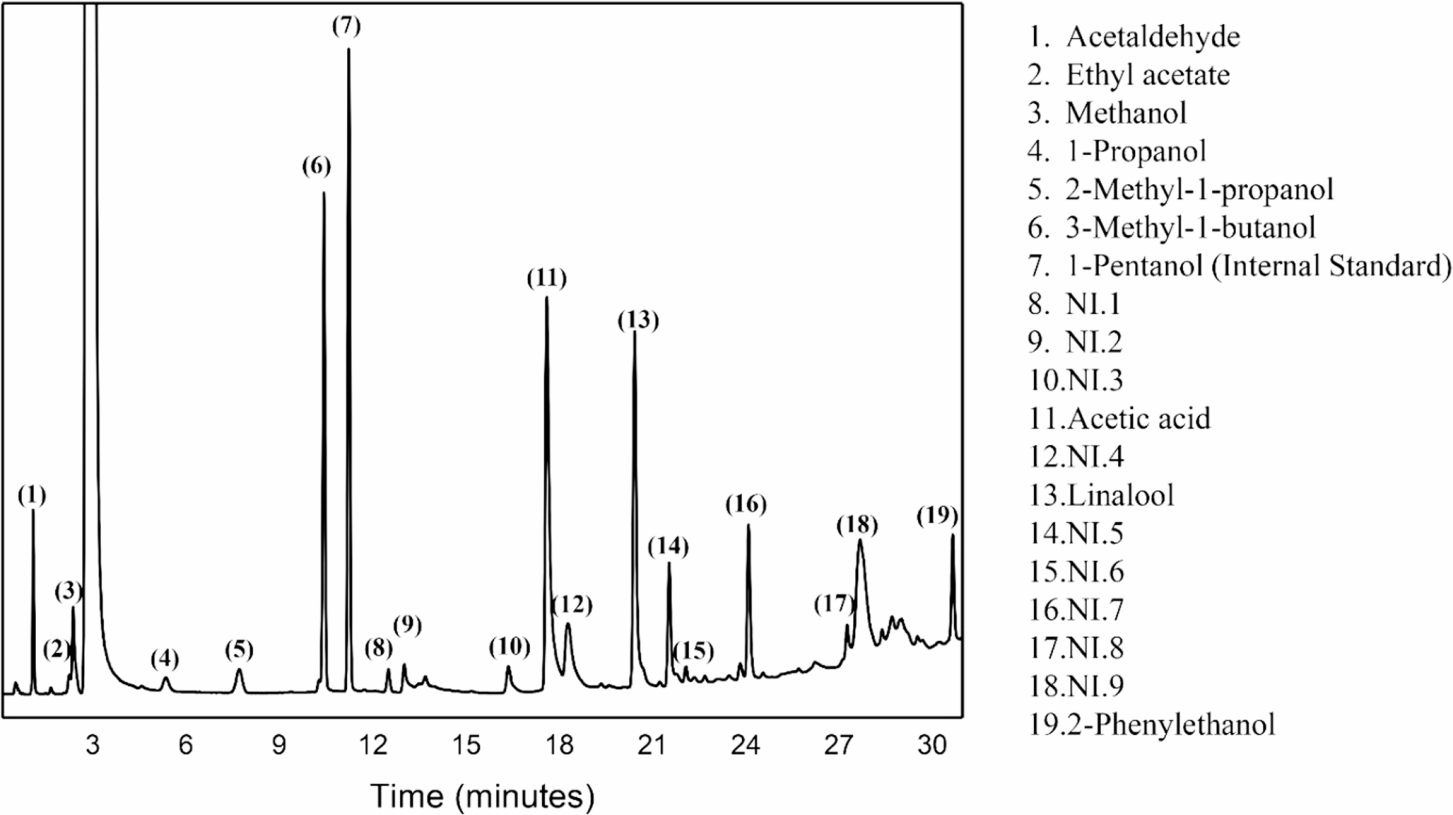
Representative GC-FID chromatogram of mead volatile compounds. Peak numbering (1–19) includes both identified compounds and unidentified peaks (NI) meeting stability criteria (CV < 10% and presence in all samples). (1) Acetaldehyde; (2) Ethyl acetate; (3) Methanol; (4) 1-propanol; (5) 2-methyl-1-propanol; (6) 3-methyl-1-butanol; (7) 1-Pentanol (Internal Standard); (8) NI.1; (9) NI.2; (10) NI.3; (11) Acetic acid; (12) NI.4; (13) Linalool; (14) NI.5; (15) NI.6; (16) NI.7; (17) NI.8; (18) NI.9; (19) 2-phenylethanol

The representative chromatographic profile showing 19 selected peaks is presented in Fig. 2.

**Table 2:** Analytical parameters for volatile compounds identified in mead by GC-FID, including retention time, calibration equations, coefficients of determination (R²), limits of detection (LOD), and limits of quantification (LOQ)

Compound	Ret. Time	Calibration Equation	*R*²	LOD (mg/L)	LOQ (mg/L)
Acetaldehyde	1.100	y = 0.0006x − 0.0124	0.9997	20.0	60.0
Ethyl acetate	2.220	y = 0.0020x − 0.0210	0.9985	6.7	20.0
Methanol	2.334	y = 0.0018x + 0.0006	0.9999	3.3	10.0
1-propanol	5.211	y = 0.0034x + 0.0100	0.9908	20.8	62.5
2-methyl-1-propanol	7.553	y = 0.0046x + 0.0061	0.9977	16.7	50.0
3-methyl-1-butanol	10.286	y = 0.0052x − 0.0859	0.9922	66.7	200.0
Acetic acid	17.656	y = 0.0017x − 0.0146	0.9971	13.3	40.0
Linalool	20.404	y = 0.0038x + 0.1370	0.9970	33.3	100.0
2-phenylethanol	30.697	y = 0.0066x + 0.0005	0.9974	6.7	20.0

### Statistical analysis

Physicochemical data were subjected to one-way ANOVA after verification of normality (Shapiro-Wilk) and homogeneity of variances (Levene), followed by Tukey’s test for mean comparison (*p* < 0.05). Results were expressed as mean ± standard deviation (*n* = 3). Pearson correlation evaluated linear relationships between extract concentration and analytical parameters. These analyses were conducted using PAST software v.4.03.

Growth curve analysis was performed in R software (version 4.3.2). Linear mixed models were fitted using the lme4 package, considering treatment, time, and treatment × time interaction as fixed effects. Effect significance was determined by Type III ANOVA with Satterthwaite method using lmerTest package. Post hoc comparisons were performed using emmeans package with Tukey adjustment (*p* < 0.05). Kinetic parameters (µmax, λ, ymax) were compared based on 95% CI, with non-overlapping intervals indicating significant difference.

Principal Component Analysis (PCA) was performed using MATLAB^®^ (version R2024b). For chromatographic data, the input matrix comprised the ratio between compound peak area and internal standard area. Odor Activity Values (OAVs) were calculated as the ratio between volatile compound concentration and its respective olfactory threshold. Data were normalized (autoscaling) prior to analysis.

## Results and discussion

### Physicochemical characteristics of jabuticaba extract and must for mead production

To evaluate the effect of jabuticaba extract supplementation on must and mead composition, physicochemical parameters were determined before (Table 3) and after fermentation (Table 4).

**Table 3:** Physicochemical characteristics of musts supplemented with Jabuticaba extract

Parameters	M0	M10	M20	M30	Pearson correlation (*r*)
pH	3.40^c^ ± 0.00	3.20^b^ ± 0.06	3.03^a^ ± 0.06	3.00^a^ ± 0.06	-0.961
Total acidity, in meq/L	20.03^a^ ± 0.31	40.13^b^ ± 0.77	60.49^c^ ± 0.73	74.03^d^ ± 0.31	0.996
Fixed acidity, in meq/L	13.74^a^ ± 0.62	30.52^b^ ± 0.59	49.60^c^ ± 0.42	69.83^d^ ± 0.73	0.999
Volatile acidity, in meq/L	6.29^a^ ± 0.54	9.61^b^ ± 0.77	10.89^b^ ± 1.12	4.20^a^ ±1.02	-0.211
Dry extract g/L	207.22^a^ ± 5.01	219.33^b^ ± 3.42	229.22^c^ ± 3.29	250.56^d^ ± 1.68	0.985
YAN, in mg N/L	47.21^a^ ± 5.07	73.51^b^ ± 2.54	127.29^c^ ± 2.54	137.15^d^ ± 3.80	0.971
Total sugars, in g/L	199.44^ab^ ± 1.10	190.32^a^ ± 13.50	196.83 ^ab^ ± 2.78	198.55^ab^ ± 3.52	0.120
Reducing sugars, in g/L	176.47^d^ ± 6.96	154.48^c^ ± 0.81	138.81^b^ ± 3.59	122.85^a^ ± 3.52	-0.996

M0: control must (0% v/v jabuticaba extract); M10, M20, and M30: musts supplemented with 10%, 20%, and 30% (v/v), respectively. Data are expressed as mean ± standard deviation (*n*=3). Different letters within the same row indicate significant differences according to Tukey’s test (*p*<0.05)

**Table 4:** Physicochemical characteristics of meads

Parameters	M0	M10	M20	M30	Pearson correlation (*r*)
pH	3.00^a^ ± 0.10	2.90^a^ ± 0.10	2.90^a^ ± 0.10	3.00^a^ ± 0.10	0.000
Total acidity, in meq/L	69.22^a^ ± 0.62	90.94^b^ ± 1.61	105.29^c^ ± 1.22	124.37^d^ ± 0.42	0.997
Fixed acidity, in meq/L	40.67^a^ ± 0.92	74.64^b^ ± 1.32	88.85^c^ ± 0.62	98.86^d^ ± 1.27	0.959
Volatile acidity, in meq/L	28.56^b^ ± 1.04	16.31^a^ ± 2.90	16.44^a^ ± 1.66	25.51^b^ ± 1.69	-0.185
Dry extract g/L	106.87^b^ ± 5.18	86.80^a^ ± 2.55	80.73^a^ ± 5.91	78.53^a^ ± 4.30	-0.911
YAN, in mg N/L	23.91^a^ ± 5.18	27.49^a^ ± 1.04	40.04^b^ ± 4.51	37.05^b^ ± 2.07	0.876
Total sugars, in g/L	87.76^b^ ± 4.30	68.31^a^ ± 1.13	67.12^a^ ± 2.45	68.07^a^ ± 3.43	-0.780
Alcohol content, in % v/v	5.60^a^ ± 0.18	8.40^b^ ± 0.22	8.29^b^ ± 0.19	8.20^b^ ± 0.09	0.735

M0: control mead (0% v/v jabuticaba extract); M10, M20, and M30: meads supplemented with 10%, 20%, and 30% (v/v), respectively. Data are expressed as mean ± standard deviation (*n* = 3). Different letters within the same row indicate significant differences according to Tukey’s test (*p* < 0.05)

The jabuticaba extract supplementation significantly modified (*p* < 0.05) the physicochemical profile of all evaluated treatments. The strong negative correlation between extract concentration and must pH (*r* = − 0.961), in contrast with the positive correlations observed for total acidity (*r* = 0.996) and fixed acidity (*r* = 0.999), confirms that jabuticaba acted as a major acidity modulator, with significant differences detected among all doses (Tukey, *p* < 0.05).

This acidification is attributable to the direct incorporation of organic acids from the fruit, whose composition has been characterized as predominantly citric acid (> 90%), followed by malic, oxalic, and tartaric acids [27, 28].

After fermentation, mead pH converged to the range of 2.90–3.00. These values are similar to those reported for meads produced with blackcurrant (*Ribes nigrum*), which ranged between 3.13 and 3.33 [29]. Despite being pronounced, this acidification may play an important technological role, as reduced pH values enhance microbiological stability [30].

Total and fixed acidity of meads maintained positive correlation with supplementation (*r* = 0.997 and *r* = 0.959, respectively), indicating retention of fruit acids combined with acid production by yeast. Although speciation was not conducted, the literature identifies succinate and lactate as relevant contributors to the non-volatile fraction under conditions of greater nutritional input [31]. Progressive increase in total acidity (61.22–94.43 meq/L) was also observed in meads supplemented with pitaya pulp [13], while genipap meads showed lower values of total acidity (49.81–53.15 meq/L) and fixed acidity (40.64–42.11 meq/L) [32].

The control must (M0) contained only 47.21 mg N/L of yeast assimilable nitrogen (YAN), confirming the well-documented nutritional deficiency of honey [4]. Extract supplementation progressively increased YAN, reaching 137.15 mg N/L in must M30. This value approaches the threshold of 140 mg N/L recommended for ensuring fermentation completion [5]. After fermentation, residual YAN ranged from 23.91 mg N/L (M0) to 40.04 mg N/L (M20) (Table 4), indicating substantial nitrogen utilization by yeast. Based on the difference between initial and final values, YAN consumption was 23.30 mg N/L (M0), 46.02 mg N/L (M10), 87.05 mg N/L (M20), and 100.10 mg N/L (M30). For comparison, consumption of 45 mg N/L was reported in unsupplemented honey musts fermented by *Torulaspora delbrueckii* and *Saccharomyces bayanus* [33]. Taken together, these results indicate that the most pronounced impact of extract addition was nutritional, as jabuticaba provided sufficient nitrogen support to address the well-documented deficiency of honey-based musts without the need for synthetic additives.

Total sugars in musts did not differ among treatments (*p* > 0.05), confirming effective standardization at 22 °Brix. In contrast, reducing sugars decreased progressively with supplementation (*r* = − 0.996), suggesting that jabuticaba extract contributed predominantly non-reducing carbohydrates. Similarly, must dry extract increased proportionally with extract addition (*r* = 0.985), reflecting the progressive incorporation of non-fermentable constituents, including organic acids and phenolic compounds [34].

These compositional and nutritional modifications translated directly into fermentative performance. Enhanced nutritional availability, particularly increased YAN levels, improved sugar-to-ethanol conversion efficiency. The control treatment (M0) achieved only limited conversion (~ 44%), yielding 5.60% v/v ethanol, whereas supplemented treatments exhibited significantly higher efficiencies (63–68%, reaching 8.20–8.40% v/v), without differences among supplemented levels (*p* > 0.05). These results are consistent with comparable improvements reported in nitrogen-supplemented sugarcane molasses fermentations [35].

The consequences of this improved conversion were reflected in final composition. Residual sugars were significantly higher in M0 (87.76 g/L) compared with supplemented treatments (67.12–68.31 g/L), which did not differ among themselves. Consistently, dry extract in meads followed an inverse trend relative to must composition (*r* = − 0.911), with the control presenting the highest value (106.87 g/L). This pattern indicates that residual material in the control consisted primarily of unconsumed sugars, whereas in supplemented treatments it corresponded mainly to the non-fermentable fruit-derived matrix.

Volatile acidity exhibited a non-linear behavior and showed no correlation with supplementation level (*r* = − 0.185), suggesting that this parameter is primarily governed by fermentative dynamics rather than by fruit addition. For comparison, pitaya meads presented volatile acidity values ranging from 10.98 to 35.60 meq/L [13], while Moscato grape meads ranged from 11.01 to 12.33 meq/L [36], placing the values observed in this study within previously reported ranges. Among treatments, the control (M0) presented the highest volatile acidity (28.56 meq/L), indicating a metabolic imbalance associated with nutritional limitation.

The mechanisms underlying this response are well established. Nitrogen scarcity is known to trigger nitrogen catabolite repression (NCR), redirecting carbon flux toward oxidized by-products [37], while deficiencies in essential cofactors may impair acetate assimilation into central metabolism, favoring volatile acid accumulation [38]. Taken together, these mechanisms support the interpretation that the elevated volatile acidity observed in M0 reflects stress-associated fermentative metabolism rather than a direct effect of fruit composition.

The reduction observed in M10 and M20 treatments indicates that jabuticaba extract acted as a complex nutritional supplement, mitigating this imbalance. Conversely, the partial recovery of volatile acidity in M30 (25.51 meq/L) suggests osmotic stress effects associated with elevated solute and organic acid concentrations, which may promote compensatory metabolic responses and increased volatile acid formation [39]. Overall, M10 and M20 treatments exhibited the most favorable metabolic balance.

Beyond improving fermentative performance, these results are consistent with previous reports indicating that jabuticaba supplementation may enhance the functional profile of fermented beverages through increased bioactive compounds and antioxidant potential [40]. Within the conditions evaluated here, jabuticaba extract acted as a multifunctional adjunct, simultaneously modulating acidity, correcting YAN deficiency, and improving fermentative efficiency, consistent with its compositional profile rich in organic acids and assimilable nitrogen.

### Monitoring of the fermentation process

#### Cell growth kinetics and fermentative dynamics

**Fig. 3:**
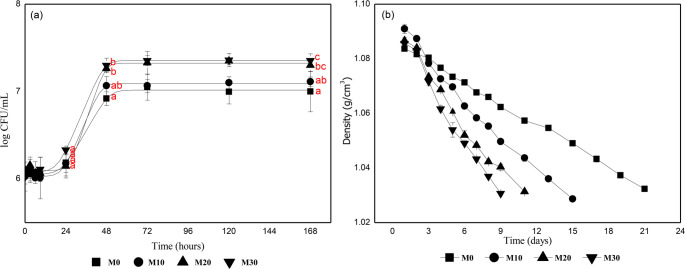
Fermentation kinetics illustrating the effect of jabuticaba extract supplementation on yeast growth and substrate consumption. (a) Growth curves of *Saccharomyces cerevisiae* M05 (log CFU/mL) fitted to the Baranyi and Roberts model. (b) Density attenuation during fermentation. Symbols: M0 (■), M10 (●), M20 (▲), and M30 (▼). Red letters in panel (a) indicate significant differences between treatments at 24, 48, and 168 h (Tukey, *p* < 0.05). Data represent mean ± standard deviation (*n* = 3)

Simultaneous monitoring of cell growth and substrate consumption revealed a strong association between nutritional availability and fermentative kinetics in *Saccharomyces cerevisiae* M05. As shown in Fig. 3, yeast population dynamics (Fig. 3a) closely paralleled the reduction in must density (Fig. 3b), highlighting the impact of supplementation on fermentation progression. Growth parameters estimated using the Baranyi and Roberts model (R² > 0.993) are summarized in Table 5.

**Table 5:** Kinetic parameters estimated from fitting the Baranyi and Roberts growth model

Samples	M0	M10	M20	M30
R²	0.993	0.996	0.998	0.998
Lag phase (λ) (h)	22.46 ± 2.14	21.92 ± 1.45	26.33 ± 1.81	18.95 ± 0.96
Maximum specific growth rate (µ) (h^− 1)^	0.035 ± 0.002	0.046 ± 0.006	0.058 ± 0.007	0.047 ± 0.004
Initial log CFU/mL	6.06 ± 0.02	6.01 ± 0.02	6.10 ± 0.01	6.07 ± 0.01
Final log CFU/mL	7.01 ± 0.02	7.09 ± 0.02	7.32 ± 0.02	7.36 ± 0.01

Values represent model-estimated parameters; statistical significance was assessed by non-overlapping 95% confidence intervals.

Standardizing the inoculum ensured a homogeneous initial cell count (~ 10⁶ CFU/mL). The absence of significant differences in initial log CFU/mL (y₀) values confirmed this uniformity, allowing subsequent kinetic differences to be attributed exclusively to the effect of supplementation. For all subsequent analyses, statistical significance was defined based on non-overlapping 95% confidence intervals (CI) of estimated parameters.

Kinetic analysis demonstrated that jabuticaba extract accelerated the exponential phase. While the control (M0) exhibited a maximum specific growth rate (µ_max_) of 0.035 h⁻¹, M20 and M30 treatments reached 0.058 h⁻¹ and 0.047 h⁻¹, respectively, differing significantly from the control. The M10 treatment (0.046 h⁻¹) showed an intermediate value, statistically positioned between the control and higher supplementation levels. These findings are consistent with the kinetic responses reported in YAN-supplemented mead fermentations, where similar values were reported by Araújo et al. [9], with an increase from 0.03 to 0.08 h⁻¹ using cowpea extract, and by Cuenca et al. [5], who obtained a µ_max_ of 0.074 h⁻¹ with 120 mg N/L of bee pollen. The consistency among these findings reinforces the role of YAN availability as the primary determinant of cell replication rate.

In addition to growth rate, cell population size was also a determining factor. As shown in Fig. 3a, clear stratification was observed in the stationary phase, with supplemented musts reaching higher plateaus. Maximum counts of M20 (7.32 log CFU/mL) and M30 (7.36 log CFU/mL) significantly exceeded the control value (7.01 log CFU/mL). Although the difference appears subtle on a logarithmic scale, it represents, in absolute terms, more than a twofold increase in viable cell numbers (from 1.0 × 10⁷ to 2.3 × 10⁷ CFU/mL). For comparison, similar increases (~ 1.32 log CFU/mL) were reported in meads supplemented with acerola pulp [11].

This larger cell population translated directly into a higher attenuation rate (Fig. 3b). The M30 treatment reached density stabilization in only 9 days, followed by M20 (11 days) and M10 (15 days). The control (M0), limited by a smaller cell population, extended fermentation to day 21 and ended with elevated density (1.032 g/cm³). This outcome indicates slow and incomplete fermentation, likely reflecting the inability of the limited cell population to sustain adequate substrate consumption rates during the stationary phase under low nutrient availability.

This trend is consistent with observations reported by Pereira et al. [6] in mead supplemented with DAP. Although the growth phase ceased early (< 48 h), the final accumulated population determined the duration of the subsequent fermentative phase. The authors observed that an increase in population from 4.2 × 10⁷ to 6.9 × 10⁷ CFU/mL reduced fermentation time from 240 h to 96 h.

Regarding higher extract proportions, M30 treatment presented significantly shorter lag phase (λ) than M20 (18.95 h vs. 26.33 h). However, despite this difference in initial adaptation, M20 and M30 converged to statistically equivalent maximum populations. This indicates that above 20% supplementation, the yeast reaches the maximum cell density supported by the medium, ensuring a cell population sufficient to sustain fermentation at maximum efficiency.

### pH kinetics during fermentation

**Fig. 4:**
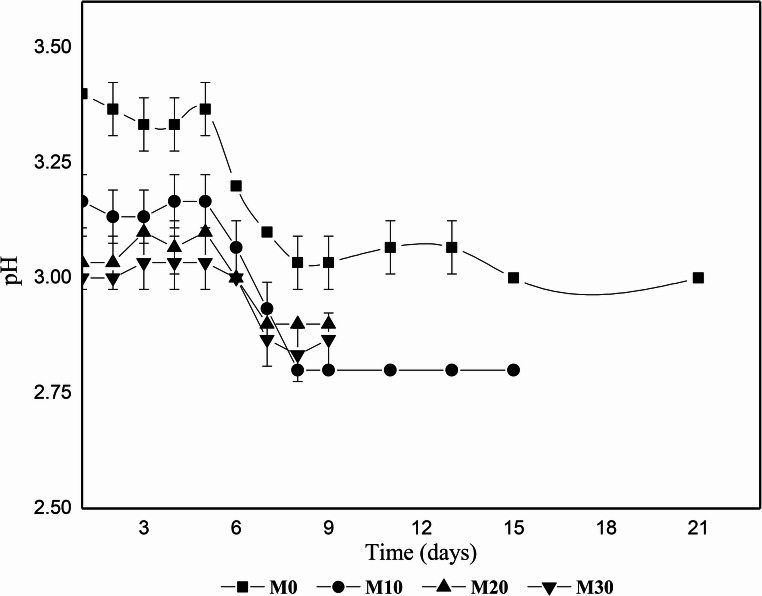
Temporal evolution of pH during mead fermentation as affected by jabuticaba extract supplementation. M0 (■), M10 (●), M20 (▲), and M30 (▼). Data represent mean ± standard deviation (*n* = 3)

pH monitoring is essential for assessing must chemical stability and yeast metabolic activity throughout the fermentative process. The temporal evolution of pH values for control mead and treatments supplemented with jabuticaba extract throughout 21 days of fermentation is presented in Fig. 4.

Jabuticaba extract supplementation significantly altered the acid-base dynamics of the must from the beginning of the process. The natural acidity of the fruit reduced the initial pH of supplemented treatments to values between 3.00 and 3.17, establishing a pre-acidified environment distinct from the control (3.40). Wanderley et al. [34] also observed that the addition of Brazilian fruit pulps to meads promoted a reduction in initial must pH, attributed to the malic and citric acid content of the fruits.

**Fig. 5:**
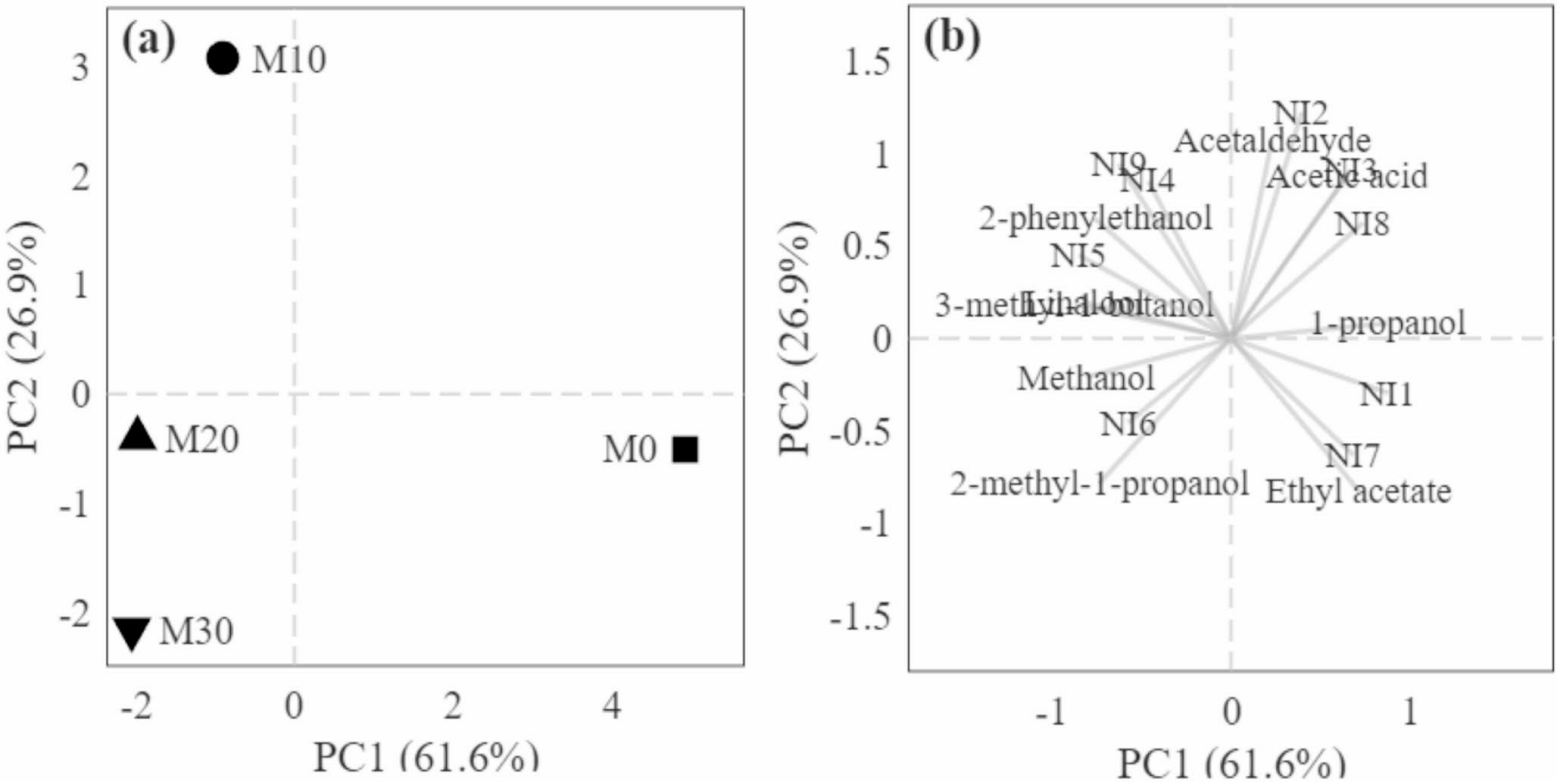
Principal component analysis (PCA) of volatile compound profiles. **a** Score plot showing sample distribution: M0 (■), M10 (●), M20 (▲), and M30 (▼). **b** Loading plot indicating variable contributions. Data were obtained from peak area ratios relative to internal standard (1-pentanol) and normalized. NI1–NI9 correspond to unidentified compounds

From a kinetic perspective (Fig. 5), a consistent triphasic behavior was observed across all treatments. Initially, a phase of relative stability occurred until day 5, suggesting that must buffering capacity was sufficient to resist initial acidification. Between days 5 and 9, a marked decrease in pH values occurred, coinciding with the period of intense fermentative activity. This profile corroborates the findings of Sroka and Tuszyński [41], who reported that organic acid biosynthesis by yeasts, notably succinic acid, intensifies during the first week of fermentation, overcoming the buffering effect of the medium.

The magnitude of total variation (Δ pH) was dependent on must composition. While the control (M0) showed a decrease of 0.40 units, M10 presented similar variation (Δ pH = 0.37), reaching the lowest final value (2.80). In contrast, M20 and M30 showed more restrained variations (Δ pH ~ 0.13), stabilizing at 2.90 and 2.87, respectively. This behavior indicates that the pre-acidification promoted by jabuticaba, combined with the enhanced buffering capacity conferred by fruit organic acids, limited the amplitude of subsequent pH reductions, maintaining the system within a physiologically adequate and stable pH range for yeast growth throughout the process.

### Influence of jabuticaba extract on the volatile compound profile of mead

#### Principal component analysis (PCA) of volatile compounds

To elucidate the global metabolic differences induced by supplementation, Principal Component Analysis (PCA) was performed using normalized areas of volatile compounds that exhibited analytical consistency (CV < 10%). This analysis accounted for 88.5% of the total variance within the first two principal components (Fig. 5).

In the score plot (Fig. 5a), the control mead (M0) was positioned in the positive region of PC1, with acetaldehyde and acetic acid presenting the highest positive loadings (Fig. 5b). This distributional pattern reflects the yeast’s metabolic response to nutritional limitation. Acetaldehyde is a central intermediate of alcoholic fermentation that, under normal conditions, exhibits a dynamic profile characterized by initial accumulation and subsequent reconsumption throughout fermentation [42]. In nitrogen-deficient musts, reconsumption tends to be less efficient, resulting in elevated residual concentrations. Acetic acid accumulation, in turn, can be attributed to redox imbalance: under nitrogen deprivation, carbon flux is diverted from biomass synthesis to acetate production as a mechanism for NADPH regeneration, an essential cofactor for stress response [43]. This behavior confirms the metabolic stress observed in the control and is consistent with the significant reduction of these by-products in supplemented fermentations [44]. Ethyl acetate, whose synthesis is negatively correlated with YAN availability [45], also contributed to the differentiation of the control treatment. Unidentified compounds NI.1, NI.2, NI.7, and NI.8 significantly contributed to control differentiation, potentially representing volatile constituents proportionally greater in the honey matrix of the control treatment, or fermentative by-products associated with YAN limitation.

In contrast, supplemented treatments (M10, M20, M30) shifted toward the negative region of PC1 (Fig. 5a), correlating with compounds of greater structural complexity, particularly the higher alcohols 3-methyl-1-butanol and 2-methyl-1-propanol. The biosynthesis of these compounds proceeds via the Ehrlich pathway, in which must-derived amino acids are transaminated to α-keto acids, decarboxylated, and subsequently reduced to their respective alcohols [46]. Specifically, 3-methyl-1-butanol derives from leucine catabolism and 2-methyl-1-propanol from valine [46, 47]. The greater availability of assimilable nitrogen in supplemented treatments (YAN from 47 to 137 mg N/L) provided the necessary precursor substrates for this pathway, resulting in a more diversified metabolic profile.

Linalool, a monoterpene not synthesized by *S. cerevisiae*, has previously been identified in jabuticaba fruits [48], confirming its varietal origin from the extract. The co-localization of NI.4, NI.5, and NI.9 with supplemented treatments in PCA space (Fig. 5b) indicates that these compounds, besides contributing to the volatile profile of mead, are associated with fruit constituent input or greater YAN availability during fermentation.

The second component (PC2, 26.9%) captured the effect of jabuticaba extract concentration and further differentiated supplemented treatments from one another. M10, positioned in the upper region of the graph, was associated with higher acetaldehyde loadings in the PCA, suggesting that this treatment represents a metabolic transition zone where fermentation was accelerated but reconsumption of this intermediate was not yet complete. In contrast, M30 was positioned in the lower region, strongly associated with methanol and NI.6. This association reflects the origin of methanol in fruit-supplemented musts, given that methanol is not produced by *S. cerevisiae* fermentative metabolism, being derived exclusively from pectin demethylation by endogenous pectin methylesterases (PME) present in the fruit [49]. The observed gradient reflects the greater incorporation of pectic material from jabuticaba peel at higher extract doses. M20 occupied an intermediate position, consistent with the balanced metabolic profile observed for this treatment across all other evaluated parameters.

#### Odor activity values (OAVs)

To estimate the sensory contribution of quantified volatile compounds, whose concentrations and odor activity values are presented in Table 6, OAVs were calculated using perception thresholds determined in hydroalcoholic matrices to minimize the bias introduced by an aqueous matrix. Compounds with OAV greater than 1 are considered active contributors to the perceived aromatic profile.

**Table 6:** Aroma descriptors, odor thresholds, concentrations (mg/L), and odor activity values (OAVs) of key volatile compounds in mead

Volatile compound	Aroma descriptor	Odor threshold (mg/L)	Concentration (mg/L)				Odor Activity Values (OAVs)				Reference
			M0	M10	M20	M30	M0	M10	M20	M30	
Acetaldehyde	Apple, nut	100	33.00	40.00	22.00	24.00	0.33	0.4	0.22	0.24	[50]
Ethyl acetate	Pineapple, fruity	7.5	21.30	14.40	15.90	17.10	**2.84**	**1.92**	**2.12**	**2.28**	[51]
Methanol	Alcoholic	> 1000	6.68	46.76	106.88	66.8	< 0.1	< 0.1	< 0.1	< 0.1	[52]
1-Propanol	Alcohol, pungent	306	15.30	15.30	15.30	9.18	0.05	0.05	0.05	0.03	[53]
2-methyl-1-propanol	Malty	40	18.40	18.40	21.20	22.00	0.46	0.46	0.53	0.55	[51]
3-methyl-1-butanol	Malty, whiskey	30	140.70	167.10	177.9	166.20	**4.69**	**5.57**	**5.93**	**5.54**	[51]
Acetic acid	Vinegar	200	884.00	566.00	698.00	590.00	**4.42**	**2.83**	**3.49**	**2.95**	[51]
2-phenylethanol	Rose, honey	10	23.40	31.30	36.40	31.70	**2.34**	**3.13**	**3.64**	**3.17**	[51]

As consistently reported in the literature, 3-methyl-1-butanol is the predominant aroma alcohol in meads [54], with elevated concentrations previously documented in fermentations conducted with *S. cerevisiae* strains [6]. In the present study, this compound stood out as the primary aromatic contributor, with OAVs ranging from 4.69 (M0) to 5.93 (M20), associated with malty and whiskey descriptors. Higher production of this alcohol has been associated with elevated YAN availability following nutritional supplementation [5], corroborating the interpretation that its biosynthesis is responsive to the nutritional enrichment provided by jabuticaba extract, an effect most pronounced in M20, which exhibited the greatest sensory impact.

2-phenylethanol was the second most impactful alcohol in the volatile profile, with OAVs between 2.34 (M0) and 3.64 (M20), conferring floral and rose notes. This compound is consistently reported as the second most abundant alcohol in meads [50], reinforcing its contribution to floral character. The literature describes 2-phenylethanol as a positive contributor to the aroma of fermented beverages, characterized by pleasant rose notes [51]. The increase observed in supplemented treatments is consistent with the higher amino acid availability in YAN-enriched musts, given that phenylalanine is the direct precursor of this alcohol via the Ehrlich pathway [34].

Ethyl acetate presented OAVs between 1.92 (M10) and 2.84 (M0), contributing fruity and pineapple notes across all treatments. Its higher concentration in control mead (21.30 mg/L) resulted in the greatest sensory impact of this ester, a behavior previously observed in fermentations conducted under nitrogen limitation [47]. Although it confers positive fruity character, excessive ethyl acetate concentrations can impart undesirable solvent-like notes [50]. The observed values remained within the range considered favorable for mead, comparable to those reported for Moscato grape meads [36] and well below levels associated with sensory defects.

Acetic acid showed OAVs between 2.83 (M20) and 4.42 (M0), indicating suprathreshold contribution across all treatments and conferring detectable vinegar-like notes. This behavior is consistent with less favorable fermentative conditions, in which nutritional limitations and osmotic stress stimulate the formation of this acid [3]. The literature reports a sensory rejection threshold for meads around 1.2 g/L [11]. In the present study, absolute concentrations ranged from 566 mg/L (M10) to 884 mg/L (M0), remaining well below this critical threshold. The significant reduction observed in M10 and M20 treatments demonstrates that the nutritional input from jabuticaba contributed to a more balanced sensory profile.

Acetaldehyde presented subthreshold OAVs in M20 and M30 (0.22–0.24), but higher values in M0 (0.33) and especially in M10 (0.40). Although it does not exceed the unitary perception threshold, elevated concentrations of this compound are generally associated with fermentations under nutritional limitations and, when excessive, can confer green apple or herbaceous notes [53]. The higher value in M10 suggests that this treatment represents a transitional sensory profile: 10% supplementation accelerated fermentation relative to the control, yet the still-limiting YAN input was insufficient to fully optimize the aromatic profile. In contrast, M20 and M30 treatments presented the lowest values, indicating a more balanced profile. The control itself (M0), despite severe nutritional limitation, presented an intermediate value, possibly due to prolonged fermentation (21 days) that allowed partial reconsumption of this intermediate over time [44].

**Fig. 6:**
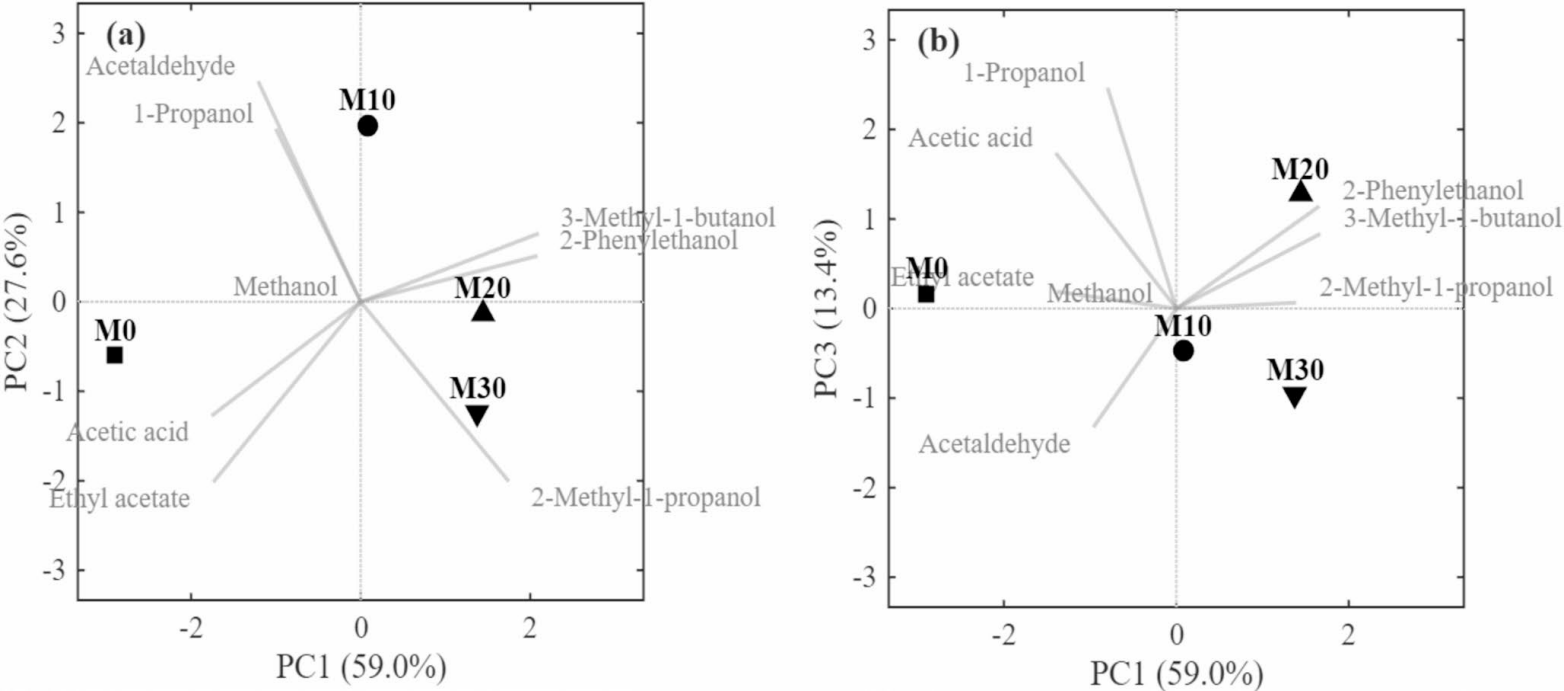
Principal component analysis (PCA) based on odor activity values (OAVs) of meads. **a** PC1 vs. PC2 score plot. **b** PC1 vs. PC3 score plot

The remaining quantified alcohols presented OAVs below 1, indicating negligible direct contribution to perceived aroma: 2-methyl-1-propanol (0.46–0.55), associated with malty notes; 1-propanol (0.03–0.05), with alcoholic and pungent descriptors; and methanol (< 0.1). Although they do not impact the sensory profile, these compounds reflect the metabolic state of fermentation. In the specific case of methanol, its quantification is mandatory from a food safety perspective, regardless of its aromatic contribution. This alcohol is toxic through two mechanisms: direct depression of the central nervous system, similar to ethanol intoxication, and metabolization to formic acid via formaldehyde, which inhibits mitochondrial cytochrome c oxidase, causing cellular hypoxia and metabolic acidosis [52]. The concentrations observed in the present study (6.68 mg/L in M0 to 106.88 mg/L in M20) remained well below the limits established by European legislation for fruit spirits (10 g/L of 100% v/v alcohol) [55]. Specifically for mead, the European Union does not establish a maximum limit for methanol, since honey musts are naturally devoid of pectins and, consequently, of this compound [52]. The presence of methanol in supplemented meads confirms its origin from the jabuticaba extract, whose pectins are hydrolyzed by endogenous pectin methylesterases during processing [56], and does not represent a toxicological risk at the observed concentrations. Integration of these data into a PCA (Fig. 6) revealed clear segregation among treatments based on olfactory quality.

In the PC1 × PC2 plane (Fig. 6a), which explained 86.6% of total variance, the first component (59.0%) clearly separated the control from supplemented treatments. Loadings indicate that M0 was characterized by higher contributions of acetic acid and ethyl acetate, while supplemented treatments were associated with higher OAVs of 2-phenylethanol and 3-methyl-1-butanol. The second component (27.6%) differentiated M10 from the other supplemented treatments, positioning it in the upper region of the graph in association with acetaldehyde and 1-propanol, consistent with its transitional profile. In the PC1 × PC3 plane (Fig. 6b), the third component (13.4%) refined the distinction between M20 and M30. M20 treatment was positioned in the upper region, associated with higher OAVs of 2-phenylethanol and 3-methyl-1-butanol, while M30 presented lower scores, consistent with its lower relative contribution of these aromatic alcohols and higher ethyl acetate content compared to M20.

Collectively, these distributional patterns confirm the aromatic transition driven by YAN availability. The control mead, characterized by intense acidic and fruity notes (acetic acid, ethyl acetate), contrasts with the floral and malty profile of supplemented treatments (2-phenylethanol, 3-methyl-1-butanol). Among all formulations, M20 achieved the optimal balance between aromatic complexity and the absence of off-flavors.

## Conclusion

Jabuticaba aqueous extract effectively corrected the nutritional deficiency of honey-based musts, enabling efficient fermentation without the use of synthetic additives. The observed shift from stress-associated volatile accumulation toward the production of desirable aromatic compounds demonstrates that YAN availability directly governs both process efficiency and aromatic quality in mead. Among the concentrations tested, 20% (v/v) provided the optimal compromise between fermentative performance and sensory complexity. These findings support the use of jabuticaba as a natural adjunct for clean-label mead production, contributing to the valorization of this underexploited native Brazilian fruit.

## Abbreviations

ANOVA: Analysis of variance
CFU: Colony-forming units
DNS − 3,5: Dinitrosalicylic acid
GC: FID-Gas chromatography-flame ionization detector
HSD: Honestly Significant Difference (Tukey)
NI: Not identified (chromatographic peaks)
OAV / OAVs: Odor activity value(s)
PCA: Principal component analysis
SCE: Sugar conversion efficiency
SD: Standard deviation
UV: Vis-Ultraviolet-visible spectrophotometry
YAN: Yeast assimilable nitrogen
YM: Yeast Malt
µmax: Maximum specific growth rate
λ: Lag phase
